# Proteomic Analysis of Vesicle-Producing *Pseudomonas aeruginosa* PAO1 Exposed to X-Ray Irradiation

**DOI:** 10.3389/fmicb.2020.558233

**Published:** 2020-12-15

**Authors:** Li Zhang, Shi-qiao Zhao, Jie Zhang, Ying Sun, Ya-liu Xie, Yan-bin Liu, Cui-cui Ma, Bo-guang Jiang, Xue-yuan Liao, Wen-fang Li, Xing-jun Cheng, Zhen-ling Wang

**Affiliations:** ^1^State Key Laboratory of Biotherapy and Cancer Center, Collaborative Innovation Center of Biotherapy, West China Hospital, Sichuan University, Chengdu, China; ^2^Department of Clinical Laboratory, Chongqing Traditional Chinese Medicine Hospital, Chongqing, China; ^3^Department of Laboratory Medicine, Sichuan Provincial People’s Hospital, University of Electronic Science and Technology of China, Chengdu, China; ^4^Department of Otolaryngology, The Seventh People’s Hospital of Chengdu, Chengdu, China; ^5^Infectious Diseases Center, West China Hospital, Sichuan University, Chengdu, China

**Keywords:** X-ray irradiation, *Pseudomonas aeruginosa* PAO1, Nucleic acid, OMVs, Proteomics, RecA, Lys

## Abstract

Ionizing irradiation kills pathogens by destroying nucleic acids without protein structure destruction. However, how pathogens respond to irradiation stress has not yet been fully elucidated. Here, we observed that *Pseudomonas aeruginosa* PAO1 could release nucleic acids into the extracellular environment under X-ray irradiation. Using scanning electron microscopy (SEM) and transmission electron microscopy (TEM), X-ray irradiation was observed to induce outer membrane vesicle (OMV) formation in *P. aeruginosa* PAO1. The size distribution of the OMVs of the irradiated PAO1 was similar to that of the OMVs of the non-irradiated PAO1 according to nanoparticle tracking analysis (NTA). The pyocin-related proteins are involved in OMV production in *P. aeruginosa* PAO1 under X-ray irradiation conditions, and that this is regulated by the key SOS gene *recA*. The OMV production was significantly impaired in the irradiated PAO1 Δ*lys* mutant, suggesting that Lys endolysin is associated with OMV production in *P. aeruginosa* PAO1 upon irradiation stress. Meanwhile, no significant difference in OMV production was observed between PAO1 lacking the *pqsR*, *lasR*, or *rhlR* genes and the parent strain, demonstrating that the irradiation-induced OMV biosynthesis of *P. aeruginosa* was independent of the *Pseudomonas* quinolone signal (PQS).

## Introduction

*Pseudomonas aeruginosa* (*P. aeruginosa)* is a gram-negative bacterium that causes global health problems. Being ubiquitous and an opportunistic pathogen, *P. aeruginosa* is now a significant source of bacteremia in hospitalized patients suffering from burn and war wound infections, chronic lung infections, and tumors (Rolston and Bodey, 1992; Bielecki et al., 2008; Calhoun et al., 2008; Yum et al., 2014). Among Caucasians, *P. aeruginosa* is currently the main cause of morbidity and mortality in cystic fibrosis and immunocompromised patients (Knapp et al., 2016; Moradali et al., 2017). In addition to both intrinsic and acquired resistance to a wide range of antibiotics, *P. aeruginosa* is able to escape detection and clearance by the host immune system during long-term colonization (Hancock and Speert, 2000; Cigana et al., 2011; Poole, 2011). In the 2019 Antibiotic Resistance Threats Report from the CDC, *P. aeruginosa* was listed as a serious pathogen, indicating an urgent need to develop new antimicrobial agents (CDC, 2019).

In contrast to the global human health needs, there is currently no satisfactory effective treatment against *P. aeruginosa* infection (Dillon et al., 2019). Furthermore, no clinically approved vaccine is currently available for application (Hoggarth et al., 2019). However, several types of vaccine candidates against *P. aeruginosa* infections are under development, including subunit vaccines, live-attenuated cell vaccines and inactivated whole vaccines (Hoggarth et al., 2019). In our previous study, we found that X-ray-inactivated *P. aeruginosa* can serve as a potential vaccine to effectively activate T cell-mediated immunity and complement-based humoral immunity *in vivo* and *in vitro* (Li et al., 2016). Irradiation-attenuated *Plasmodium falciparum* sporozoite vaccines are currently undergoing investigation in phase III clinical trials for human malaria (Richie et al., 2015). In addition, intranasal vaccination with the irradiated influenza A virus vaccine (c-Flu) has been reported to activate cross-protective immunity (Alsharifi et al., 2009; David et al., 2017). Unlike traditional methods such as heat killing and chemicals, ionizing irradiation can penetrate pathogens and destroy nucleic acids without protein structure destruction (Kaminski et al., 2014; Reber et al., 2016). Moreover, the immunogenicity of pathogen proteins can be retained after irradiation inactivation, indicating that irradiation might be a potential tool for preparation of whole-cell inactivated vaccines (Seo, 2015). However, the overall biological effects of bacteria exposed to X-ray irradiation are still unclear and required further investigation.

In this study, we aimed to investigate the biological changes in the laboratory *P. aeruginosa* PAO1 strain after exposure to X-ray irradiation. Our results revealed that X-ray irradiation can induce *P. aeruginosa* PAO1 to release nucleic acid into their extracellular environment. Of note, we showed that PAO1 exposed to X-ray radiation can produce OMVs with nucleic acids, for which we propose the name irradiation-induced OMVs (IOMVs). Our data further demonstrated that the IOMV production in PAO1 was associated with pyocin production and was mediated by *recA*, a key gene of the SOS system. Regulation of this process was related to Lys, but may be independent of the *Pseudomonas* quinolone signal (PQS).

## Materials and Methods

### Bacterial Strains, Plasmids, and Primers

The bacterial strains and plasmids used in this study are shown in Supplementary Table S1. The primers used in this study are shown in Supplementary Table S2.

### Materials

1-*N*-phenyl-2-naphtylamine (NPN) and HEPES were obtained from Sigma-Aldrich (St Louis, MO, United States). Ethylenediaminetetraacetic acid (EDTA) and citric acid were purchased from Kelong (Chengdu, China). Polymyxin B was purchased from Meilunbio (Dalian, China). A stock solution of NPN (0.5 M) was prepared in acetone and diluted to a final concentration of 40 mM using 5 mM HEPES buffer (pH 7.2) for the fluorometric assays. The preparation of 5 mM HEPES buffer proceeds as follows: dissolve 1.1915 g HEPES (Sigma-Aldrich, United States) in about 800 mL of double- deionized H_2_O and adjust pH to 7.2 with 1 M NaOH solution while stirring. Add deionized H_2_O to the total volume to 1 L and filter sterilized [0.22-μm-pore-size filters (Millipore)].

### Culture Preparation

The frozen bacterial stocks of the strains used were subcultured onto LB agar plates (BD Biosciences, Heidelberg, Germany) from a single colony for 16–18 h at 37°C. Overnight cultures were inoculated to an optical density at OD_600_ of 0.05 and incubated to exponential phase (OD_600_ = 1.0 ± 0.1) in LB medium with shaking (220 rpm) at 37°C. Bacterial cells were centrifuged (5,316 × *g*, 20 min) and suspended in 0.9% saline (Kelong, Chengdu, China) to adjust the OD_600_ to 50. Colony-forming unit (CFU) counting was performed by serial dilution (Gupta et al., 1996).

### X-Ray Irradiation

X-ray irradiation of *P. aeruginosa* and *Escherichia coli* K12 was performed as described previously with some modifications (Li et al., 2016). The bacterial suspensions were prepared as mentioned above and the samples were divided evenly into two parts: one without irradiation was conducted as a control, and the other was exposed to X-ray irradiation using a RS2000 Biological X-ray irradiator (Rad Source Technologies, FL, United States) at 160 kV/25 mA. We added a layer of copper to minimize the biological effects of low-energy irradiation on the bacterial cells.

### Determination of Bacterial Survival and Extracellular Nucleic Acids

The bacterial suspensions were exposed to X-ray irradiation from 0 Gy to 2.24 kGy at a dose rate of 7.086 Gy/min. Samples were taken every 0.14 kGy. The surviving fraction was calculated as the number of viable bacteria divided by the total CFU (Gupta et al., 1996). After filtration through 0.45 μm-pore-size filters (Millipore), the nucleic acid concentration of the filtrates was measured by using a NanoDrop 2,000 and the extracellular nucleic acids was determined by agarose gel electrophoresis. After irradiation, the bacterial suspensions were placed at 4°C and the concentration of extracellular nucleic acids was measured every 24 h. In addition, the supernatants of the control and the irradiated PAO1 (under 0.98 kGy) were collected and evenly divided into three parts: (1) digestion with 10 μg/ml DNase I (Qiagen, Germany); (2) digestion with 100 μg/ml RNase I (Qiagen, Germany); and (3) no treatment. After the digestion reactions, the samples were immediately assessed by agarose gel electrophoresis and the grayscale image was analyzed by ImageJ 1.51K software. All assays were conducted as soon as possible to ensure the reliability of the data.

### Confocal Laser Scanning Microscopy (CLSM)

After exposure to X-ray irradiation at the dose of 0.98 kGy, the *P. aeruginosa* PAO1 suspensions were immediately stained with TOTO^TM^-1 (2 μM) and FM^TM^4-64 (5 μg/ml) for 5 min to label the nucleic acid and the membrane, respectively. A 2 μl droplet of cells were observed on a 1.2% agarose pad with N-STORM/A1 microscope (Nikon) at 488 nm and 561 nm. Image analysis was performed using NIS-ELEMENTS AR software (Nikon, Japan).

### Scanning Electron Microscopy (SEM)

The SEM samples of *P. aeruginosa* PAO1 and *E. coli* K12 were prepared as described previously with some modifications (Koon et al., 2019). Briefly, after exposure to X-ray irradiation at 0.98 kGy (PAO1) or 1.26 kGy (K12), the bacterial cells were centrifuged at 3,000 × *g* for 10 min. The pellets were washed three times in 0.1 M PBS (pH 7.2) and fixed in a 2.5% glutaraldehyde solution at 4°C overnight. Then, the cells were dehydrated using a graded ethanol series (30, 50, 70, 80, 90, and 100% × 3) at 10 min per step. The dehydrated samples were subjected to critical point drying with liquid carbon dioxide for 1 h. The samples were covered with gold-palladium by sputter coating to prevent charging in the microscope. The images were collected using a Field Emission Scanning Electron Microscope JEOL JSM–7500F.

### Transmission Electron Microscopy (TEM)

The TEM samples of *P. aeruginosa* PAO1 cells and OMVs were prepared as described previously (Stukalov et al., 2008; Dosunmu et al., 2015). For the bacterial samples, ultrathin sections were stained with 2% uranyl acetate and lead citrate, and were sent to the Center of Forecasting and Analysis of Sichuan University (Sichuan, China) for imaging with a TEM (H-600, Hitachi, Japan). For the OMVs, 20-μl droplets of vesicle suspensions were placed onto carbon-coated 200-mesh copper grids. The samples were then stained with a 1% (w/v) phosphotungstate acid solution for 5 min. The redundant fluid was removed with a piece of filter paper. The grids were allowed to air dry and were then imaged with a TEM (JEM-2,100 Plus, Japan).

### OMV Isolation

After *P. aeruginosa* was grown to the exponential phase (OD_600_ = 1.0 ± 0.1), the culture preparation was the same as that described above and were divided evenly into two groups: one group without irradiation was kept as a control group, and the other group were exposed to X-ray irradiation at a dose of 0.98 kGy. The IOMVs and the control OMVs were isolated based on a previous protocol with some modifications (Kadurugamuwa and Beveridge, 1995). Briefly, the bacterial suspensions were centrifuged (8,000 × *g*, 30 min, 4°C), and the supernatant was sequentially centrifuged (16,000 × *g*, 30 min, 4°C) to clear most of the contaminating cell debris (Metruccio et al., 2016). Then, the resulting supernatant was filtered through 0.45 μm-pore-size filters (Millipore), and the OMVs were pelleted from the filtrates by ultracentrifugation (100,000 × *g*, 2 h, 4°C) in a Ti 32 rotor (Beckman Instruments, OPTIMA XE-90, Canada). The pellets containing OMVs were suspended in 1 ml MV buffer. The preparation of MV buffer proceeds as follows: dissolve 50 mM Tris, 5 mM NaCl, and 1 mM MgSO_4_ in about 800 mL of deionized H_2_O and adjust pH to 7.4 with 1 M HCl solution while stirring. Add deionized H_2_O to the total volume to 1 L and filter sterilized [0.22-μm-pore-size filters (Millipore)]. OMV aliquots (10 μl) were spread onto LB agar plates at 37°C for one day to assess sterility, and the remaining OMVs were stored at −80°C until use. The same method was used to isolate the OMVs of *E. coli* K12.

To isolate OMVs from the culture supernatants of *P. aeruginosa* PAO1 and PA14, PAO1 and PA14 were cultivated in 200 ml of LB medium with shaking to an OD_600_ of 1.0 ± 0.1, and then the bacterial cells were removed by centrifugation (5,316 × g, 20 min). The supernatant was filtered through 0.45-μm-pore-size filters (Millipore), and the resulting filtrates were centrifuged (16,000 × g, 30 min, 4°C) to clear most of the contaminating cell debris. Then, the OMVs were pelleted from the supernatant by ultracentrifugation (100,000 × g, 2 h, 4°C). The pellets containing OMVs were suspended in 200 μl MV buffer. OMV aliquots (10 μl) were spread onto LB agar plates at 37°C for 1 day to assess sterility, the remaining OMVs were stored at −80°C until use.

### Nanoparticle Tracking Analysis (NTA)

The size distribution and concentration of OMVs were measured by nanoparticle tracking analysis (NTA, Particle Metrix, Germany) and analyzed using Zetaview Analysis software (Vestad et al., 2017). Briefly, OMV samples were thawed over ice and diluted in distilled water (1:200–1:4,000). One milliliter of diluted sample was injected for quantification of the OMV size distribution and concentration using Zetaview software with the following specific analysis parameters: maximum particle size: 2,000, minimum particle size 0. All measurements were performed at room temperature (24.5^*o*^C ± 0.1).

### Protein Extraction and Trypsin Digestion

Total protein was extracted from bacterial suspensions using lysis buffer (8 M urea, 1% Protease Inhibitor Cocktail III) (PTM Bio, Zhejiang, China). The samples of the control and irradiated *P. aeruginosa* PAO1 (0.98 kGy) were sonicated three times on ice using a high-intensity ultrasonic processor (Scientz, Zhejiang, China) in lysis buffer. The remaining cell debris was removed by centrifugation at 12,000 × *g*, 4^*o*^C for 10 min. The resulting supernatants were collected and the protein concentration was quantified using a BCA kit (Beyotime).

For digestion, the protein solution was reduced with 5 mM DTT for 30 min at 56°C and alkylated with 11 mM iodoacetamide (IAA, Sigma-Aldrich, Germany) for 15 min at room temperature in the dark. Then, protein samples were diluted by adding 100 mM TEAB to urea at a concentration less than 2 M. Finally, trypsin (Promega, WI, United States) was added at 1:50 (trypsin: protein) mass ratio for the first digestion overnight and 1:100 (trypsin: protein) mass ratio for a second 4 h digestion.

### TMT Labeling and LC-MS/MS Analysis

After trypsin digestion, the tryptic peptide mixtures were desalted by using a Strata X C18 SPE column (Phenomenex, CA, United States) and vacuum-dried. Peptides were reconstituted in 0.5 M TEAB and processed with a TMT kit (Thermo Fisher Scientific, United States) according to the manufacturer’s protocol. Briefly, one unit of TMT reagent was thawed and reconstituted in acetonitrile (ACN, Sigma-Aldrich, Germany). Then, the peptide mixtures were incubated for 2 h at room temperature, pooled, desalted, and dried by vacuum centrifugation. One microgram of dried peptide was required to determine labeling efficiency.

Peptides were dissolved in 0.1% formic acid (FA, Sigma-Aldrich, Germany) and separated using an EASY-nLC 1,000 UPLC system (Thermo Fisher Scientific, United States). Mobile phase A was water containing 0.1% formic acid/2% acetonitrile; mobile phase B was acetonitrile containing 0.1% formic acid/90% acetonitrile. The peptides were separated by using an ultra-high-performance liquid phase system and then were injected into an NSI ion source for ionization and analyzed by Orbitrap Lumos mass spectrometry. The ion source voltage was at 2.0 kV.

### Database Searches and Bioinformatics Analysis

The resulting MS/MS data were processed using the Maxquant search engine (v.1.5.2.8) at Jingjie PTM Biolab (Hangzhou, China). Mass spectrometry data were searched with the Mascot 2.3 (Matrix Science) and matched against the UniProt database. At the same time, the mass spectrometry results were evaluated using a reverse database search method to exclude the false positive rate (FDR). Trypsin/P was specified as cleavage enzyme, allowing up to two missed cleavages. The minimal peptide length was set to 4 amino acids and the maximum charge state was set to 5. Peptides precursor ions were searched with a maximum mass deviation of 10 ppm and fragment ions with a maximum mass deviation of 0.02 Da. Cysteine alkylation was set as a fixed modification and the Met oxidation was set as variable modifications.

Subcellular localization was predicted using Wolfpsort software. Change in protein abundance with a corrected *p* < 0.05 were considered significant. The false discovery rate (FDR) was set to < 1%, and a minimum score for the peptides was set > 40. The raw data in this study have been deposited into the ProteomeXchange Consortium with the dataset identifier PXD019035.

### NPN Uptake Assays

The permeability properties of the bacterial outer membrane were determined using the NPN uptake assays as described previously (Helander and Mattila-Sandholm, 2000a). Briefly, after exposure to X-ray irradiation with 0.98 kGy, the irradiated PAO1 cells and the non-irradiated PAO1 cells were washed three times and resuspended in 5 mM HEPES buffer (pH 7.2), and the suspensions were adjusted to OD_600_ = 0.5. Aliquots (100 μl) of the suspensions were immediately pipetted into the fluoroplate plates (four parallel wells/sample) containing NPN (10 μM) to a total volume of 200 μl. EDTA (1.0 mM, pH 7.2), polymyxin B (10 μM), and citric acid (10 mM, pH 4.0) were used as positive substances in our study (Helander and Mattila-Sandholm, 2000a,b; Alakomi et al., 2006). The black fluorotitre plate was then subjected to fluorometry utilizing the automated fluorometer Fluoroskan Ascent FL (LabSystems). The fluorescence intensity values of four parallel wells of each sample was recorded within 3 min using standard Fluoroskan filters for 350 nm (excitation) and 415 nm (emission). The NPN uptake factor was calculated as the ratio of background-subtracted fluorescence intensity values of the bacterial suspension and of the HEPES buffer according to a previous study (Helander and Mattila-Sandholm, 2000a).

### Construction of Mutants in *P. aeruginosa* PAO1

PAO1 mutants were constructed following a previously described protocol (Hmelo et al., 2015). Briefly, the upstream and downstream regions of each gene open reading frame (ORF) were amplified from PAO1 genomic DNA, digested with *Kpn*I/*Eco*RI, and ligated into the linearized suicide vector pEX18Gm. The resulting mixtures were transformed into the competent *E. coli* DH5α cells by electroporation. The constructed recombinant plasmids pEX18Gm-*recA*, pEX18Gm-*lys*, pEX18Gm-*prtN*, pEX18Gm-*PA0634*, pEX18Gm-*PA3866*, and pEX18Gm-*PA0985* were mobilized from *E. coli* S17.1 into PAO1 by mating. The single colonies were streaked onto non-salt LB (NSLB) plus 15% sucrose (Sigma-Aldrich) plates and incubated at 30°C for sucrose counterselection. Finally, the sucrose-resistant colonies that grew on LB agar and *Pseudomonas* Isolation Agar (PIA) plates (BD, Germany) but not on LB agar plates containing 30 μg/ml gentamicin were selected, and the desired mutation was confirmed by PCR and sequencing (Tsingke, Beijing, China). All primers used in this study are listed in Supplementary Table S2.

### Alamar Blue Assay

The metabolic activity of PAO1 and the Δ*recA* mutant after exposure to X-ray irradiation was assayed by the Alamar Blue assay (Li et al., 2016). Briefly, 10^6^ CFU/ml of bacterial cells were incubated with 10 μl Alamar Blue dye in a 96-well plate at 37°C for 4 h. The medium without cells was used as a negative control. The fluorescence values were monitored at 560 nm (excitation) and 590 nm (emission).

### Statistical Analysis

The results of bacterial survival and nucleic acid concentration measurements were analyzed using OriginPro Graphing software (Version 9.1, OriginLab Corporation, MA). The results of the OMV production were analyzed using GraphPad Prism 7.0 software for Windows (GraphPad Software Inc., La Jolla, CA, United States) The grayscale images were analyzed by Image J 1.51K software. Statistical significance was evaluated using Student’s *t*-test, one-way ANOVA, or two-way ANOVA. *p* < 0.05 represents statistically significant differences.

## Results

### The Effect of X-Ray Irradiation on *P. aeruginosa* PAO1

**FIGURE 1:**
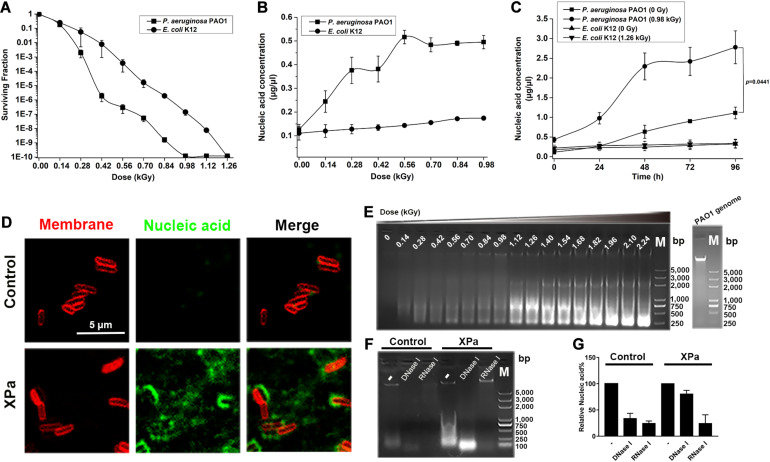
The effect of X-ray irradiation on *P. aeruginosa* PAO1. **(A)** The surviving fraction of PAO1 under X-ray irradiation. **(B)** Extracellular nucleic acid concentration of PAO1 exposed to different doses of irradiation. **(C)** Determination of the extracellular nucleic acid concentrations of PAO1 over 96 h after exposure to X-ray irradiation. **(D)** Micrographs of the irradiated PAO1 cells (XPa) and the non-irradiated PAO1 cells (Control) stained with the membrane dye FM^TM^4-64 (red) and the DNA stain TOTO^TM^-1 iodide (green). Green-stained nucleic acids were observed with XPa. **(E)** The extracellular nucleic acids of PAO1 after exposure to different doses of irradiation were detected by agarose gel electrophoresis. **(F)** The supernatant of PAO1 was digested by DNase I and RNase I, and analyzed by agarose gel electrophoresis. Lane 1, the supernatant of the control; lane 2, the supernatant of the control with DNase I digestion; lane 3, the supernatant of the control with RNase I digestion; lane 4, the supernatant of the irradiated PAO1; lane 5, the supernatant of the irradiated PAO1 with DNase I digestion; lane 6, the supernatant of the irradiated PAO1 with RNase I digestion; lane M, DNA ladder. **(G)** Grayscale analysis of the ratio of DNA and RNA in the nucleic acids shown in **(F)**.

We investigated the effect of X-ray irradiation on the viability of *P. aeruginosa* PAO1 by employing the irradiation-sensitive *E. coli* K12 strain as a control. A previous study showed that *E. coli* lacks mechanisms to avoid the lethal effects of DNA double-strand breaks by ionic irradiation (Cox and Battista, 2005). As shown in Figure 1A, compared with K12, the surviving fraction of PAO1 decreased sharply in the range of 0.14–0.42 kGy. At dose of 0.28 kGy, 99.9% of PAO1 cells lost viability, while for K12, a dose of approximately 0.56 kGy was required to achieve the same effect. The bacterial viability was abrogated at 0.98 kGy for PAO1 but at 1.26 kGy for K12. This result suggested that compared with *E. coli* K12, *P. aeruginosa* PAO1 is more sensitive to X-ray irradiation.

To examine whether nucleic acids were present in the extracellular environment, we measured the extracellular nucleic acid concentrations of PAO1 and K12 at various doses of irradiation. As shown in Figure 1B, the extracellular nucleic acid concentration of PAO1 increased gradually with increasing irradiation doses; however, transient stability in the range of 0.28–0.42 kGy was noted, which was consistent with the dose required for a sharp decrease in the surviving fraction. The nucleic acid reached the saturation at around 0.56 kGy. Interestingly, compared with PAO1, K12 rarely released nucleic acids under X-ray irradiation (Figure 1B). We then monitored the extracellular nucleic acid concentrations of PAO1 and K12 over 96 h after irradiation. The results showed that the nucleic acid release of the irradiated PAO1 (0.98 kGy) increased significantly compared with that of the control group (0 Gy) (Figure 1C). However, there was no significant increase in nucleic acid release for *E. coli* K12 under X-ray irradiation (Figure 1C). Moreover, we labeled the membrane with FM^TM^4-64 (red) and the nucleic acids with TOTO^TM^-1 (green). Compared with those of the cells without irradiation, many green-stained nucleic acids of the irradiated PAO1 cells diffused into the extracellular environment (Figure 1D).

The supernatants of the irradiated PAO1 at different doses were separated and examined by agarose gel electrophoresis. The results showed that there were variously sized nucleic acid fragments in the supernatant and the largest fragment was equal to the size of the genome (Figure 1E). When the doses reached to 1.12 kGy, the size distribution of the nucleic acids was mainly concentrated at 750 bp, while the size distribution was mainly concentrated at 250 bp following further dose increase (Figure 1E). To determine whether the nucleic acids were DNA or RNA, we then digested the supernatants of the irradiated PAO1 and the control with DNase I and RNase I, respectively. Agarose gel electrophoresis showed that the bands were significantly weakened, suggesting that the extracellular nucleic acids contain both DNA and RNA (Figure 1F). Grayscale analysis demonstrated that the nucleic acids released by the irradiated PAO1 contained more RNA (Figure 1G). Collectively, these results showed that unlike *E. coli* K12, X-ray irradiation could induce *P. aeruginosa* PAO1 to release nucleic acids, especially RNA.

### IOMV Production in *P. aeruginosa* PAO1 Under X-Ray Irradiation

**FIGURE 2:**
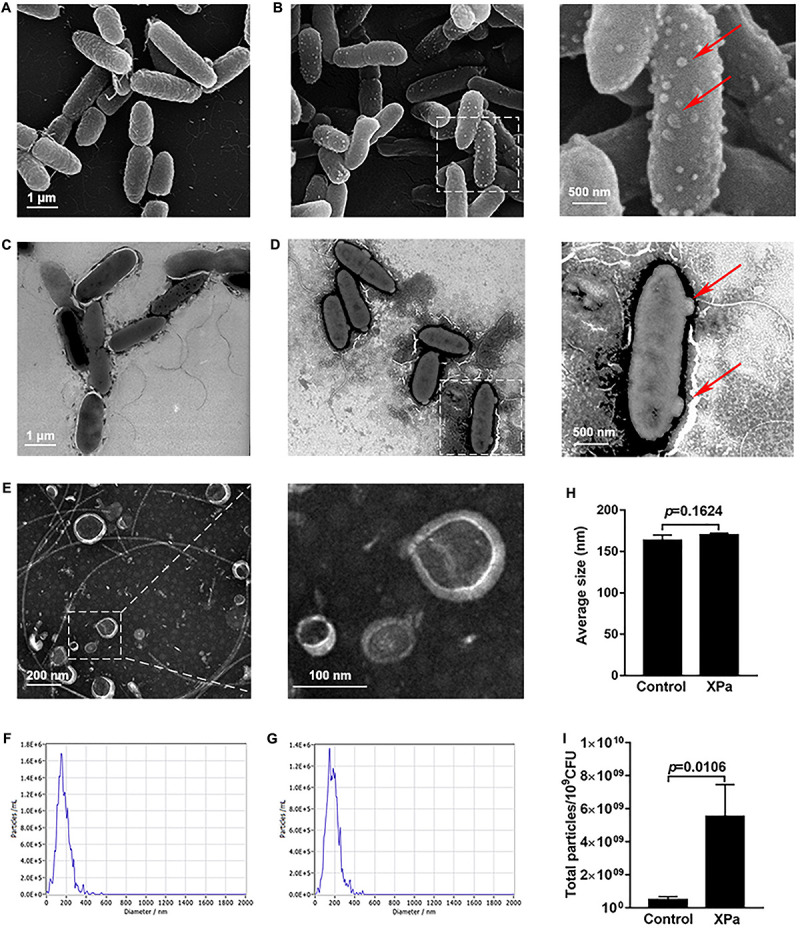
The IOMV production of *P. aeruginosa* PAO1 under X-ray irradiation conditions. **(A)** Scanning electron microscope image of PAO1 without irradiation. **(B)** Scanning electron microscope image of PAO1 after exposure to X-ray irradiation. **(C)** Transmission electron microscope image of PAO1 without irradiation. **(D)** Transmission electron microscope image of PAO1 after exposure to X-ray irradiation. The red arrows represent the particles on the cell surface of the irradiated PAO1. **(E)** Transmission electron microscope image of IOMVs. **(F)** Size distribution of OMVs from the non-irradiated PAO1 as measured by NTA. **(G)** Size distribution of IOMVs from the irradiated PAO1 as measured by NTA. **(H)** The average sizes of OMVs from the non-irradiated PAO1 (Control) and irradiated PAO1 (XPa). **(I)** Quantification of the OMV yields of PAO1 with (XPa) and without (Control) irradiation as measured by NTA. Error bars represent the standard deviation from 3 independent assays, and *p* < 0.05 represents statistically significant differences.

The morphological and structural alterations of *P. aeruginosa* PAO1 after exposure to X-ray irradiation were examined by SEM and TEM. The results showed many spherical nanoscale particles on the cell surface of the irradiated PAO1 compared with control group (Figures 2B,D). We then isolated the particles by ultracentrifugation and observed by TEM. As shown in Figure 2E, these particles purified from the irradiated PAO1 are spherical structures and similar to OMV sizes. Furthermore, NTA was performed to characterize the OMVs, and the data suggested that compared with that of the control OMVs, no significant differences existed in the size distribution of the IOMVs (Figures 2G–I). Furthermore, the yields of IOMVs were significantly higher than the amount of OMVs produced by the non-irradiated PAO cells (Figure 2J). In addition, we also examined the morphology and OMV production of *E. coli* K12 under irradiation stress. The result showed that X-ray irradiation did not induce OMV formation in *E. coli* K12 (Supplementary Figure 1).

Many studies have described the presence of nucleic acids in OMVs (Renelli et al., 2004; Fulsundar et al., 2014; Bitto et al., 2017). We extracted OMVs from the same volume of supernatant of control and irradiated PAO1 and performed agarose gel electrophoresis, as shown in Supplementary Figure 2. The result revealed that IOMVs of PAO1 carried a large amount of nucleic acids. Together, these results suggested that X-ray irradiation can induce *P. aeruginosa* PAO1 to produce IOMVs carrying nucleic acids.

### Proteomic Analysis of *P. aeruginosa* PAO1 Under X-Ray Irradiation

Previous studies reported that OMV formation is associated with pyocin production in *P. aeruginosa* under stress conditions (Toyofuku et al., 2014; Turnbull et al., 2016). To examine whether the abundance of pyocin protein changed after exposure to X-ray irradiation, we performed a quantitative proteomics analysis comparing protein abundance between whole-cell lysates from the irradiated and non-irradiated *P. aeruginosa* PAO1. At a *p*-value < 0.05, a greater than 2-fold increase is represented a higher abundance threshold, while a less than 1/2-fold decrease indicated a lower abundance threshold. The result showed that the abundance of 48 protein increased and of 8 decreased under X-ray irradiation stress (Supplementary Figure 3A). Subcellular localization prediction revealed that among the 48 high abundance proteins, 56.25% were mainly located in the periplasmic regions and 33.33% were in cytoplasmic regions (Supplementary Figure 3B). As expected, several proteins involved in pyocin synthesis were found to be present at higher abundance levels, including PrtN, PA0634, PA0985, PA3866, PA0621, and PA0646 (Table 1), suggesting that the abundance of pyocin protein changed in *P. aeruginosa* PAO1 exposed to X-ray irradiation stress.

**TABLE 1:** The abundance of proteins changed significantly in *P. aeruginosa* PAO1 under X-ray irradiation.

UniProt accession	Locus tag	Protein name	Protein abundance^*a*^	Protein abundance^*b*^	Fold change^*c*^	Protein function
**Proteins with increased abundance**						
Q9I706	PA0126		1.656	0.368	4.50	Uncharacterized protein
Q9I6I2	PA0309		1.476	0.682	2.164	Uncharacterized protein
Q9I6H7	PA0314	FliY	1.594	0.786	2.028	L-cysteine transporter of ABC system
Q06552	PA0610	PrtN	1.249	0.562	2.222	Transcription regulatory protein
G3XD08	PA0621		1.646	0.61	2.698	Hypothetical/phage related
G3XD68	PA0634		1.747	0.288	6.066	Hypothetical/phage related
Q9I5S0	PA0646		0.798	0.389	2.051	Hypothetical/phage related
Q9I5D1	PA0807	AmpDh3	1.42	0.391	3.632	Peptidoglycan binding-like protein
Q9I585	PA0856		1.761	0.434	4.058	Uncharacterized protein
P72161	PA0869	PbpG	0.818	0.4	2.045	D-alanyl-D-alanine endopeptidase
Q9I550	PA0907	AlpA	1.204	0.437	2.755	Uncharacterized protein
Q9I547	PA0910	AlpD	1.024	0.353	2.901	Uncharacterized protein
Q9I505	PA0953		1.215	0.439	2.768	Probable thioredoxin
Q9I4Y4	PA0985	PyoS5	1.363	0.347	3.928	Pyocin S5
Q9I4G6	PA1171	SltB2	1.677	0.419	4.002	Probable lytic murein transglycosylase
Q9I4B6	PA1222	MltA	1.367	0.524	2.609	Membrane-bound lytic murein trans-glycosylase A
Q9I457	PA1287		1.577	0.733	2.151	Glutathione peroxidase
Q9I398	PA1624		1.455	0.658	2.211	Uncharacterized protein
Q9I379	PA1646		1.539	0.583	2.64	Probable chemotaxis transducer
Q9I2N2	PA1863	ModA	1.699	0.575	2.955	Molybdate-binding periplasmic protein
Q9I271	PA2044		1.338	0.635	2.107	Uncharacterized protein
Q9I106	PA2476	DsbG	1.466	0.71	2.065	Thiol:disulfide interchange protein
Q9I0S3	PA2562		1.591	0.703	2.2562	Uncharacterized protein
Q9I0H1	PA2667	MvaU	1.832	0.713	2.569	Uncharacterized protein
Q9I0A1	PA2742	RpmI	1.243	0.179	6.944	50S ribosomal protein L35
Q9HZZ0	PA2854		1.662	0.608	2.73	Uncharacterized protein
Q00514	PA3101	XcpT	1.293	0.567	2.28	Type II secretion system protein G
Q9HZ35	PA3205		1.249	0.355	3.52	Uncharacterized protein
Q59641	PA3227	PpiA	1.665	0.536	3.106	Peptidyl-prolyl cis-trans isomerase A
Q9HYY3	PA3257	Prc	1.625	0.467	3.48	Periplasmic tail-specific protease
Q9HXW5	PA3675		1.918	0.442	4.339	Uncharacterized protein
Q9HXU8	PA3692	LptF	0.836	0.327	2.56	Lipotoxin F
G3XD17	PA3737	DsbC	1.504	0.744	2.022	Thiol:disulfide interchange protein
Q9HXE0	PA3866	PyoS4	1.38	0.451	3.06	Pyocin S4
Q9HX76	PA3940		1.527	0.535	2.86	Probable DNA binding protein
Q9HWE8	PA4250	RpsN	0.911	0.395	2.306	30S ribosomal protein S14
Q9HWE3	PA4255	RpmC	1.86	0.28	3.496	50S ribosomal protein L29
G3XD15	PA4552	PilW	1.242	0.586	2.12	Type 4 fimbrial biogenesis protein
Q9HVA8	PA4687	HitA	1.679	0.587	2.86	Ferric iron-binding periplasmic protein
Q9HTX3	PA5217	AfuA	1.659	0.64	2.59	Probable binding protein component of ABC iron transporter
Q9HTP9	PA5305		1.581	0.412	3.83	Uncharacterized protein
Q9HTN9	PA5315	RpmG	1.86	0.28	6.643	50S ribosomal protein
Q9HTM5	PA5330		1.828	0.488	3.75	Uncharacterized protein
Q9HTL0	PA5348	HupA	0.869	0.381	2.281	DNA-binding protein HU-alpha
Q9HTF0	PA5414		1.308	0.616	2.12	Uncharacterized protein
Q9HTC5	PA5441		1.582	0.658	2.40	Uncharacterized protein
Q9HT98	PA5472		1.685	0.508	3.32	Uncharacterized protein
**Proteins with decreased abundance**						
Q9I710	PA0122	RahU	0.528	1.684	0.314	Lipid binding protein
Q9I494	PA1244		0.665	1.642	0.405	Uncharacterized protein
Q9HXP9	PA3745	RpsP	0.595	1.364	0.436	30S ribosomal protein S16
P46384	PA4080	PilG	0.733	1.55	0.473	Twitching motility protein
Q9HWF8	PA4240	RpsK	0.411	1.055	0.39	30S ribosomal protein S11
Q9HWD4	PA4264	RpsJ	0.639	1.406	0.454	30S ribosomal protein S10
Q9HWD0	PA4268	RpsL	0.368	0.983	0.374	30S ribosomal protein S12
Q9HT11	PA5563	Soj	0.354	1.487	0.238	Chromosome partitioning protein

**^*a*^The abundance of proteins in the irradiated P. aeruginosa PAO1; ^*b*^The abundance of proteins in the non-irradiated P. aeruginosa PAO1; ^*c*^The ratio of protein abundance in P. aeruginosa PAO1 with and without X-ray irradiation.**

**FIGURE 3:**
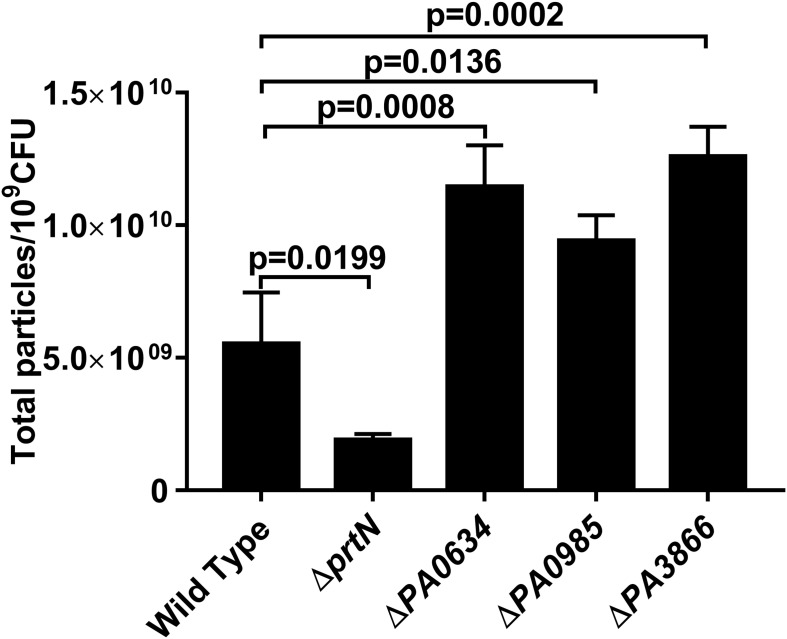
IOMV production is affected by mutations in pyocin production in *P. aeruginosa* PAO1 under X-ray irradiation conditions. Data are expressed as the total particle counts per 10^9^ colony forming units (Total particles/10^9^CFU), and the error bars represent the standard deviation from 3 independent assays. *P* < 0.05 was considered to indicate statistically significant differences.

To determine whether pyocin protein is associated with IOMV production in *P. aeruginosa* PAO1 under X-ray irradiation conditions, we constructed a series of gene-deficient mutants (Δ*prtN*, Δ*PA0634*, Δ*PA0985*, and Δ*PA3866*). With X-ray irradiation exposure, the IOMV yields of the Δ*prtN* mutant were significantly reduced, while those of the Δ*PA0634*, Δ*PA0985*, and Δ*PA3866* mutants were significantly increased (Figure 3). These results suggested that *prtN* positively regulates IOMV production, and that PA0634, PA0985 and PA3866 are involved in IOMV production in *P. aeruginosa* after exposure to X-ray irradiation.

### Assessment of the Outer Membrane Integrity in the Irradiated *P. aeruginosa* PAO1

To assess the outer membrane integrity of PAO1 exposed to X-ray irradiation, the uptake of 1-*N*-phenylnaphthylamine (NPN) was determined. NPN is a non-polar probe that strongly fluoresces when entering the phospholipid layer. EDTA, polymyxin B and citric acid can disrupt the outer membrane of gram-negative bacteria and were used as positive controls in this study. The data are shown in Table 2. EDTA (1 mmol l^–1^), polymyxin B (10 μmol l^–1^), and citric acid (10 mmol l^–1^) brought about a significantly higher NPN uptake than that of control cells, indicating that the outer membrane of PAO1 was damaged when treated with these chemicals. Of note, the irradiated PAO1 cells significantly increased NPN uptake, thus indicating that the outer membrane of *P. aeruginosa* PAO1 exposed to X-ray irradiation could be damaged.

**TABLE 2:** Fluorescence intensity values obtained from 1-N-phenylnaphthylamine (NPN) uptake assay in *P. aeruginosa* PAO1 under X-ray irradiation.

Sample	NPN	Fluorescence intensity values (mean ± SD)	Fluorescence intensity values after background subtraction^*a*^	NPN uptake factor^*b*^
Buffer	−	5.7E + 04 ± 2.9E + 04		
	+	1.1E + 05 ± 3.4E + 04	5.3E + 04 ± 5.0E + 03	1
Control Cells	−	1.38E + 05 ± 3.2E + 04		
	+	3.43E + 05 ± 6.3E + 04	2.05E + 05 ± 3.1E + 04	3.85 ± 0.24
Cells + EDTA (1 mmol l^–1^)	_	8.83E + 04 ± 3.3E + 04		
	+	5.18E + 05 ± 1.48E + 05	4.30E + 05 ± 1.15E + 05*	8.02 ± 1.46*
Cells + Polymyxin B (10 μmol l^–1^)	_	1.45E + 05 ± 1.5E + 04		
	+	5.33E + 05 ± 1.3E + 04	3.88E + 05 ± 2E + 03*	7.36 ± 0.64*
Cells + citric acid (10 mmol l^–1^)	_	1.19E + 05 ± 5.4E + 04		
	+	4.05E + 05 ± 6.5E + 04	2.86E + 05 ± 1.1E + 04*	5.42 ± 0.30*
Irradiated PAO1 Cells	_	1.93E + 05 ± 7.3E + 04		
	+	6.0E + 05 ± 8.0E + 04	4.07E + 05 ± 7E + 03**	7.72 ± 0.58*

^*a*^The difference of fluorescence intensity values before/after adding NPN for each sample. ^*b*^NPN uptake factor represents the ratio of background-subtracted fluorescence intensity values of the bacterial suspension and of the 5 mM HEPES buffer, respectively. *p < 0.05 compared with control cells; **p < 0.01 compared with control cells.

### The Effect of RecA and Lys on IOMV Production in *P. aeruginosa* PAO1 Under X-Ray Irradiation

**FIGURE 4:**
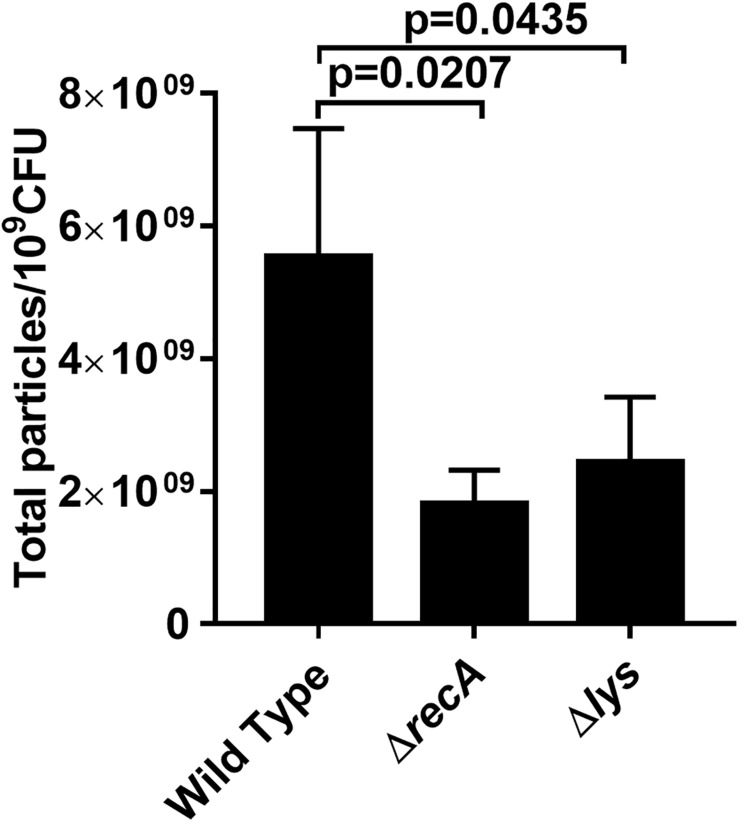
Effects of RecA and Lys on IOMV production in PAO1 after exposure to X-ray irradiation. Data are expressed as the total particle counts per 10^9^ colony forming units (Total particles/10^9^CFU), and the error bars represent the standard deviation from 3 independent assays. *P* < 0.05 was considered to indicate statistically significant differences.

A previous study reported that the gene *prtN* is positively regulated by the RecA-mediated SOS response (Matsui et al., 1993). Therefore, we studied whether a relationship exists between RecA and IOMV production in PAO1 after exposure to X-ray irradiation. DNA-damaging agents such as ciprofloxacin are reported to induce *P. aeruginosa* to produce more OMVs in a RecA-dependent manner (Maredia et al., 2012; Turnbull et al., 2016). In our study, we found that under X-ray irradiation conditions, the IOMV yields of the Δ*recA* mutant decreased about 60% compared with that of the wild-type strain (Figure 4). To exclude the possibility that X-ray irradiation impairs the metabolic activity in the Δ*recA* mutant, we performed an Alamar Blue assay and observed that the deletion of the *recA* gene did not affect the metabolic activity in PAO1 under X-ray irradiation (Supplementary Figure 4). This result suggested that RecA mediates IOMV formation in PAO1 under X-ray irradiation conditions. The R- and F-pyocin gene cluster encodes Lys (PA0629), which is an endolysin that can degrade peptidoglycan to release pyocins (Turnbull et al., 2016). Our proteomics analysis also revealed some peptidoglycan-associated proteins were affected by X-ray irradiation (AmpDh3, Prc, and PpiA) (Table1). We deleted the *lys* gene of PAO1, and found that the IOMV yields of the Δ*lys* mutant decreased about 56% compared to that of the parent strain after exposure to X-ray irradiation (Figure 4). We next searched the amino acid sequence of *E. coli* K12 for homologs of the RecA and Lys (PA0629) encoded by *P. aeruginosa* PAO1 and found that *E. coli* K12 harbors RecA (71%), which has roles in homologous recombination, DNA repair, and the induction of the SOS response. In addition, the glycoside hydrolase family 19 protein, which is encoded by gene *FAZ84_09245* of *E. coli* K12, exhibits 45% homology with the Lys (PA0629) of PAO1. However, in our study, X-ray irradiation did not induce *E. coli* K12 to release nucleic acids and produce OMVs (Figure 1A and Supplementary Figure 1), suggesting that these two proteins may not initiate nucleic acid release and OMV production in *E. coli* K12 under irradiation stress. Taken together, these results suggested that under X-ray irradiation conditions, RecA and Lys are involved in IOMV production in *P. aeruginosa*.

### IOMV Production in *P. aeruginosa* Is Independent of the PQS

**FIGURE 5:**
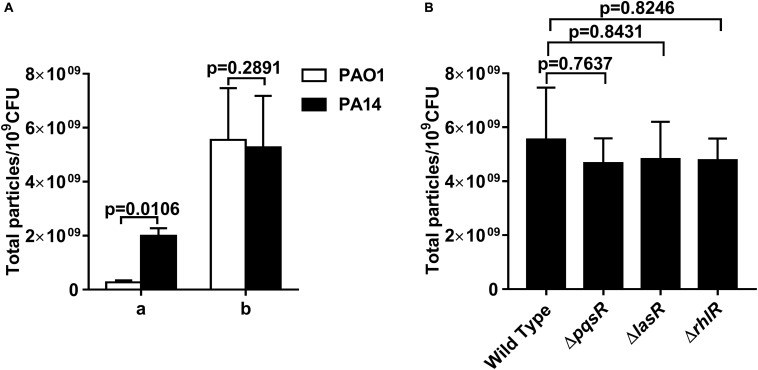
OMV production in *P. aeruginosa* is independent of the PQS. **(A)** Comparison of OMV yields between PAO1 and PA14. a, the OMVs from the 200-ml cultures of exponentially growing PAO1 and PA14. b, the IOMVs from the same volume supernatant of irradiated PAO1 and PA14. **(B)** Effects of PqsR, LasR and RhlR on IOMV production in PAO1 after exposure to X-ray irradiation. Data are expressed as the total particle counts per 10^9^ colony forming units (Total particles/10^9^CFU), and the error bars represent the standard deviation from 3 independent assays. *P* < 0.05 represents statistically significant differences.

We examined whether the IOMV production in *P. aeruginosa* was related to the PQS, which is an important quorum-sensing molecule. The movement of PQS out of the inner membrane has been reported to be crucial for OMV production in *P. aeruginosa* (Florez et al., 2017); however, other studies have found that PQS is not required under some stress conditions (MacDonald and Kuehn, 2012; Toyofuku et al., 2014; Turnbull et al., 2016). To explore the effect of the PQS on IOMV biosynthesis in *P. aeruginosa* under X-ray irradiation, we first utilized two common laboratory-adapted strains (PAO1 and PA14) with significant differences in PQS membrane distribution (Florez et al., 2017). As shown in Figure 5A, the OMV production from the culture supernatant of PA14 was higher than that of PAO1, which is consistent with previous results (Florez et al., 2017). However, after exposure to X-ray irradiation, no defect in the IOMV yields of PAO1 and PA14 was found (Figure 5A). There are three known quorum sensing (QS) systems in *P. aeruginosa*, namely, *las*, *rhl*, and *pqs* (Wilder et al., 2011). PqsR (MvfR) is a transcriptional regulator of the *pqs* system that can bind to PQS (Lu et al., 2012). We quantified the amount of IOMVs produced by the irradiated PAO1 Δ*pqsR* mutant and found that deletion of *pqsR* did not affect the IOMV production of PAO1 under X-ray irradiation (Figure 5B). We further assessed the IOMV yields of the two other QS system mutants of PAO1, Δ*lasR* and Δ*rhlR*, after exposure to X-ray irradiation. The *lasR* and *rhlR* genes positively regulate PQS production in *P. aeruginosa* (Venturi, 2006). The results showed that there were no significant differences in IOMV production between the mutants and the parent strain PAO1 (Figure 5B). Together, these results suggested that IOMV production appears to be independent of the PQS in *P. aeruginosa* under X-ray irradiation.

## Discussion

In this study, we investigated physiological changes in *P. aeruginosa* PAO1 under X-ray irradiation conditions. Morphological observations revealed that X-ray irradiation induces PAO1 to produce IOMVs. Pyocins are associated with IOMV production in *P. aeruginosa* PAO1 under X-ray irradiation stress. RecA and Lys are involved in the IOMV production in *P. aeruginosa* PAO1, while the PQS is not involved in this process.

Vaccination is arguably considered the most important prevention strategy against infections. Ionic irradiation has been reported to be a promising approach for the development of highly effective vaccines due to better preservation of the immunogenicity of pathogen proteins (Magnani et al., 2009; David et al., 2017). Our previous study showed that X-ray-inactivated *P. aeruginosa* can serve as a potential vaccine to effectively activate cellular immunity and humoral immunity *in vivo* and *in vitro* (Li et al., 2016). It is well known that safety is crucial to vaccine development, and our study indicated that at a dose of 0.98 kGy, no live bacterium was detectable at any time point, suggesting that 0.98 kGy is the minimal lethal dose for completely inactivating *P. aeruginosa* PAO1.

Nucleic acids are the primary target of ionizing irradiation-induced cellular damage (Trampuz et al., 2006). Our study showed for the first time that *P. aeruginosa* PAO1 released its nucleic acids into the extracellular environment shortly after exposure to X-ray irradiation (Figure 1B). This phenomenon was not observed for *E. coli* K12, which released hardly any nucleic acid under X-ray irradiation. This suggests that *E. coli* K12 and *P. aeruginosa* may have distinct physiological mechanism to respond to irradiation stress. Previous studies have reported that the presence of extracellular nucleic acids may be attributed to cell lysis (Hamilton et al., 2005; Thomas et al., 2008) or active secretion systems (Allesen-Holm et al., 2006), or may be associated with OMVs (Turnbull et al., 2016). Using SEM and TEM, we observed many OMVs attached to the cell surface of the irradiated PAO1 (Figure 2). Furthermore, we found that these OMVs produced by the irradiated PAO1 could carry nucleic acids (Supplementary Figure 2), which is consistent with previous studies demonstrating that bacteria can produce OMVs carrying DNA and RNA (Maredia et al., 2012; Gamalier et al., 2017; Toyofuku et al., 2019). Many studies have shown that bacterial OMVs are immunomodulators that stimulate humoral and cell-mediated immune responses (Roberts et al., 2008; Park et al., 2010; Kim et al., 2013). For example, *E. coli*-derived OMVs efficiently prevented bacterium-induced inflammatory response syndrome (Kim et al., 2013). OMVs of *Bordetella pertussis* combined with alum adjuvants could confer protection against pertussis in a mouse model (Roberts et al., 2008). In *P. aeruginosa*, OMVs can initiate the immune response of host cells (Bauman and Kuehn, 2006; Zhang et al., 2018). Our study showed that X-ray irradiation induced an increase in the IOMV production in PAO1, but the detailed mechanism remains unclear. Thus, it is necessary to explore the regulatory mechanism of IOMV formation in *P. aeruginosa*, which may be helpful to develop IOMV-based vaccines in the future.

Pyocin production is reported to be involved in OMV biosynthesis (Maredia et al., 2012; Toyofuku et al., 2014; Turnbull et al., 2016). The proteomics results in this study demonstrated that the abundance levels of some pyocin proteins substantially increased in *P. aeruginosa* PAO1 exposed to X-ray irradiation (Table 1). PrtN has been reported to be the master regulator of the pyocin genes and can positively activate pyocin production (Toyofuku et al., 2014). In our study, we found a high abundance level of PrtN in PAO1 after exposure to X-ray irradiation, and the IOMV yields of PAO1 Δ*prtN* were significantly reduced upon irradiation stress. Thus, IOMV formation can be reasonably argued to be associated with pyocin production in *P. aeruginosa* PAO1 under X-ray irradiation conditions. Three types of pyocins (R, F, and S types) exist in *P. aeruginosa*; PA0985 and PA3866 belongs to the S-type pyocin family, and PA0634 is an F-type pyocin member (Elfarash et al., 2014; Turnbull et al., 2016). Pyocin S5 encoded by *PA0985* can cause membrane damage to *P. aeruginosa* and result in leakage of intracellular materials, such as nucleic acids (Ling et al., 2010). However, our results showed that the IOMV yields of the Δ*PA0985* mutant significantly increased, implying that PA0985 has another regulation function related to IOMV production in *P. aeruginosa* under X-ray irradiation conditions. Deletion of genes *PA3866* (pyocin S4) and *PA0634* (pyocin F2) also increased IOMV production in PAO1 upon irradiation stress, but the detailed mechanism of how PA3866 and PA0634 regulate IOMV production in *P. aeruginosa* under X-ray irradiation conditions requires further study.

In most bacteria, RecA is the key regulator of the SOS response and it is activated upon stresses such as UV radiation and oxidation (Zeynep and Didier, 2014; Maslowska et al., 2019). Our results showed that the IOMV production of the PAO1 Δ*recA* mutant was substantially impaired, suggesting that RecA is involved in the IOMV biosynthesis of *P. aeruginosa* PAO1 under X-ray irradiation conditions. A recent study reported that the RecA-mediated SOS response regulates the endolysin Lys, which is encoded by the R- and F-pyocin gene cluster, upon exogenous stresses (Turnbull et al., 2016). Lys can degrade cell wall peptidoglycan, resulting in weakening of its connection with the outer membrane and thereby causing explosive cell lysis and OMV formation in *P. aeruginosa* (Turnbull et al., 2016). Although the proteomics analysis did not detect the abundance change of Lys, the IOMV yields of the PAO1Δ*lys* mutant were significantly reduced. These results indicated that in addition to IOMVs formed by membrane blebbing, explosive cell lysis mediated by Lys endolysin is associated with IOMV production in *P. aeruginosa* PAO1 under X-ray irradiation conditions. The quorum-sensing PQS plays an essential role in stimulating OMV formation in *P. aeruginosa* (Mashburn-Warren et al., 2009; Florez et al., 2017). Florez et al. reported that the PQS can insert into the outer membrane of *P. aeruginosa*, inducing membrane curvature and subsequent OMV release (Florez et al., 2017). However, previous studies have shown that stress-induced OMV production can occur in a PQS-independent manner (MacDonald and Kuehn, 2012; Toyofuku et al., 2014; Turnbull et al., 2016). Similar results were also found in our study: upon X-ray irradiation stress, no significant difference in IOMV production was observed between PAO1 strains lacking the *pqsR*, *lasR*, or *rhlR* genes, and the parent strain (Figure 5B), suggesting that the PQS may not be involved in the IOMV production in *P. aeruginosa*.

To the best of our knowledge, this is the first study to determine the presence of extracellular nucleic acid and IOMV formation in *P. aeruginosa* PAO1 exposed to X-ray irradiation. Furthermore, we showed that pyocins involved in the IOMV production in PAO1 under irradiation stress. This stress-induced biogenesis process was associated with the key SOS gene, *recA*, and the endolysin gene, *lys*. Our study offers new insights into the biological effect of ionizing irradiation on *P. aeruginosa* and the pathways that regulate IOMV formation.

## Data Availability Statement

The available datasets were analyzed in this study and can be found via ProteomeXchange with identifier PXD019035.

## Author Contributions

Z-lW, LZ, S-qZ, and JZ conceived and designed the experiments. LZ, JZ, YS, C-cM, B-gJ, X-yL, W-fL, and X-jC performed the experiments. LZ, S-qZ, JZ, YS, C-cM, and W-fL analyzed the data. S-qZ, Y-lX, Y-bL, C-cM, and JZ contributed reagents, materials, and analysis tools. LZ and Z-lW wrote the manuscript. Z-lW, Y-bL, and JZ provided project funding. All authors have reviewed and approved the final manuscript.

## Conflict of Interest

The authors declare that the research was conducted in the absence of any commercial or financial relationships that could be construed as a potential conflict of interest.
