# Supplementary material for: Proteomic Analysis of Vesicle-Producing Pseudomonas aeruginosa PAO1 Exposed to X-Ray Irradiation
Source: Front Microbiol. 2020 Dec 15;11:558233. doi: 10.3389/fmicb.2020.558233 (PMC7770229; doi:10.3389/fmicb.2020.558233)
Supplement: Supplementary file 1 [file Data_Sheet_1.docx]

Supplementary Material

**Table S1.** Bacterial strains and plasmids used in this study.

| ***E. coli* strain** | **Genotype** | | **Source or reference** |
| --- | --- | --- | --- |
| DH5α | *F^–^ Φ80lacZΔM15 Δ(lacZYA-argF) U169 recA1 endA1 hsdR17 (rK–, mK+) phoA supE44 λ– thi-1 gyrA96 relA1 tonA* | | Invitrogen |
| S17.1 | *Thi-1 thr leu tonA lac Y supE recA::RP4-2-Tc::Mu Km^R^* | | (Simon et al., 1983) |
| K12 | *F^–^ λ– ilvG– rfb-50 rph-1* | | ATCC |
| ***P. aeruginosa* strain** | | **Genotype or serotype** | **Source or reference** |
| *P. aeruginosa* PAO1 | *Pseudomonas aeruginosa* PAO1 | | Our laboratory |
| *P. aeruginosa* PA14 | *Pseudomonas aeruginosa* PA14 | | Our laboratory |
| *P. aeruginosa* PAO1 Δ*pqsR* | *Pseudomonas aeruginosa* PAO1 Δ*pqsR* | | This study |
| *P. aeruginosa* PAO1 Δ*lasR* | *Pseudomonas aeruginosa* PAO1 Δ*lasR* | | This study |
| *P. aeruginosa* PAO1 Δ*rhlR* | *Pseudomonas aeruginosa* PAO1 Δ*rhlR* | | This study |
| *P. aeruginosa* PAO1 Δ*lys* | *Pseudomonas aeruginosa* PAO1 Δ*lys* | | This study |
| *P. aeruginosa* PAO1 Δ*recA* | *Pseudomonas aeruginosa* PAO1 Δ*recA* | | This study |
| *P. aeruginosa* PAO1 Δ*prtN* | *Pseudomonas aeruginosa* PAO1 Δ*prtN* | | This study |
| *P. aeruginosa* PAO1 Δ*PA0634* | *Pseudomonas aeruginosa* PAO1 Δ*PA0634* | | This study |
| *P. aeruginosa* PAO1 Δ*PA0985* | *Pseudomonas aeruginosa* PAO1 Δ*PA0985* | | This study |
| *P. aeruginosa* PAO1 Δ*PA3866* | *Pseudomonas aeruginosa* PAO1 Δ*PA3866* | | This study |
| **Plasmid** | **Genotype** | | **Source or reference** |
| pEX18GM | Gm^R^, *oriT*, *sacB*, *lacZa*, suicide vector with MCS from pUC18 | | (Hoang et al., 1998) |
| pEX18GM-*lys* | *lys* deletion suicide vector | | This study |
| pEX18GM-*recA* | *recA* deletion suicide vector | | This study |
| pEX18GM-*prtN* | *prtN* deletion suicide vector | | This study |
| pEX18GM-*PA0634* | *PA0634* deletion suicide vector | | This study |
| pEX18GM-*PA0985* | *PA0985* deletion suicide vector | | This study |
| pEX18GM-*PA3866* | *PA3866* deletion suicide vector | | This study |

Km^R^, kanamycin resistance; Gm^R^, gentamycin resistance.

**Table S2**. Primers used in this study.

| **Primer** | **Organism** | | **Sequence (5’-****3’)** |
| --- | --- | --- | --- |
| *lys* up P1 | | *Pseudomonas aeruginosa* PAO1 | 5’**-**CCGGAATTCAGGTTCATCTCGGCTATGCC-3’ |
| *lys* up P2 | | *Pseudomonas aeruginosa* PAO1 | 5’-CCATTCAGGGGTCAGTTTCATCGATCCTCCTG-3’ |
| *lys* down P1 | | *Pseudomonas aeruginosa* PAO1 | 5’**-**AACTGACCCCTGAATGGCCAGGCGGAG-3’ |
| *lys* down P2 | | *Pseudomonas aeruginosa* PAO1 | 5’**-**CGGGGTACCTTGTCGGATGCAAACGAAAAAG-3’ |
| *recA* up P1 | | *Pseudomonas aeruginosa* PAO1 | 5’**-**CCGGAATTCAGCAGAGAGACCTTGAGTGGC-3’ |
| *recA* up P2 | | *Pseudomonas aeruginosa* PAO1 | 5’**-**CATTGGCCTAGGGCGCGCTTCTTGTTCTC-3’ |
| *recA* down P1 | | *Pseudomonas aeruginosa* PAO1 | 5’**-**GCGCGCCCTAGGCCAATGGCGATCGTG-3’ |
| *recA* down P2 | | *Pseudomonas aeruginosa* PAO1 | 5’**-**CGGGGTACCACGCGGTGAAGGAAATCGC-3’ |
| *prtN* up P1 | | *Pseudomonas aeruginosa* PAO1 | 5'-CCGGAATTCTGCTGGAACCGCTGCATC-3' |
| *prtN* up P2 | | *Pseudomonas aeruginosa* PAO1 | 5'-ATGCGATGCTGTCAGGGTATTCCCTCCTGCGGC-3' |
| *prtN* down P1 | | *Pseudomonas aeruginosa* PAO1 | 5'-GCAGGAGGGAATACCCTGACAGCATCGCATCCTGA-3' |
| *prtN* down P2 | | *Pseudomonas aeruginosa* PAO1 | 5'-CGGGGTACCGAAGATCCGCGCCATGGA-3' |
| *PA0634* up P1 | | *Pseudomonas aeruginosa* PAO1 | 5'-CCGGAATTCAGCGTTGTCGAGCCGGGA-3' |
| *PA0634* up P2 | | *Pseudomonas aeruginosa* PAO1 | 5'-GGTTGACCTCACCGACTACGCGCTCCTCAAGCC-3' |
| *PA0634* down P1 | | *Pseudomonas aeruginosa* PAO1 | 5'-TTGAGGAGCGCGTAGTCGGTGAGGTCAACCGGG-3' |
| *PA0634* down P2 | | *Pseudomonas aeruginosa* PAO1 | 5'-CGGGGTACCGCAATCAGTGCCGAACTGTC-3 |
| *PA0985* up P1 | | *Pseudomonas aeruginosa* PAO1 | 5'-CCGGAATTCATCGAAGACGAAGTCCATCGAC-3' |
| *PA0985* up P2 | | *Pseudomonas aeruginosa* PAO1 | 5'-AGGCCTTGCTTGGTTTTAGACTTCTCCATTGGTGAGTGT-3' |
| *PA0985* down P1 | | *Pseudomonas aeruginosa* PAO1 | 5'-AATGGAGAAGTCTAAAACCAAGCAAGGCCTCATTAAAC-3' |
| *PA0985* down P2 | | *Pseudomonas aeruginosa* PAO1 | 5'-CGGGGTACCGCTTCGGCGTCTCTACTCA-3’ |
| *PA3866* up P1 | | *Pseudomonas aeruginosa* PAO1 | 5'-CCGGAATTCATAGACCGTACTGGAGGAAACG-3' |
| *PA3866* up P2 | | *Pseudomonas aeruginosa* PAO1 | 5'-AGTTAGGATTGATCAGAATGGCTTTCCTATCGAAAGAGTT-3' |
| *PA3866* down P1 | | *Pseudomonas aeruginosa* PAO1 | 5'-ATAGGAAAGCCATTCTGATCAATCCTAACTCTGTCGAAATA-3' |
| *PA3866* down P2 | | *Pseudomonas aeruginosa* PAO1 | 5'-CGGGGTACCATCAGGCCATCGTACTCCTG-3 |

**Supplementary Figures**


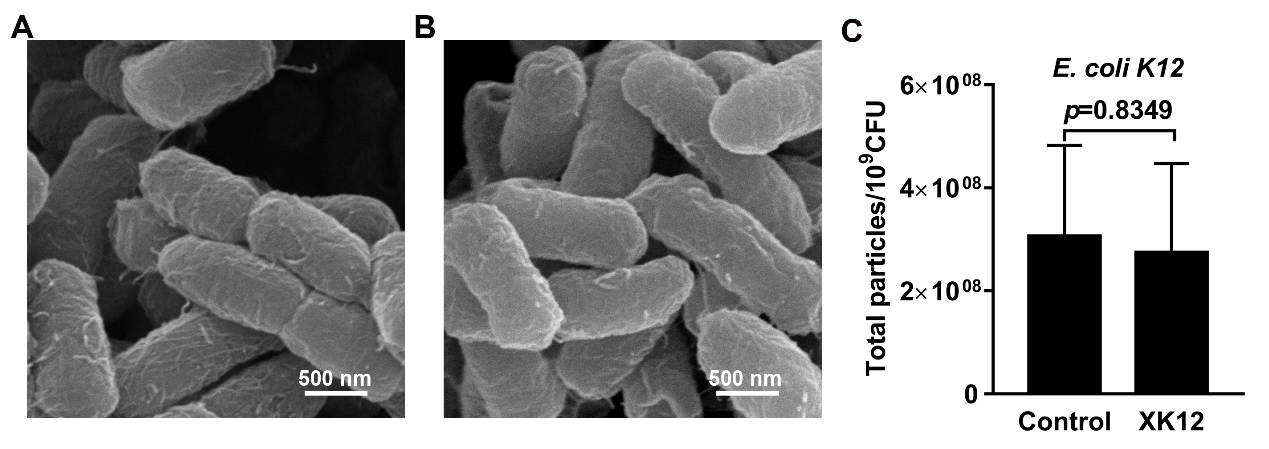


**Supplementary Figure 1.** X-ray irradiation did not induce OMV production in *E. coli* K12. Scanning electron microscope image of **(A)** non-irradiated *E. coli* K12 (Control) and **(B)** irradiated *E. coli* K12 (XK12). **(C)** Quantification the yields of OMVs produced by Control and XK12. Data are expressed as the total particle counts per 10^9^ colony forming units (Total particles/10^9^CFU), error bars represent the standard deviation from 3 independent assays. *p* < 0.05 was considered to indicate statistically significant differences.

**
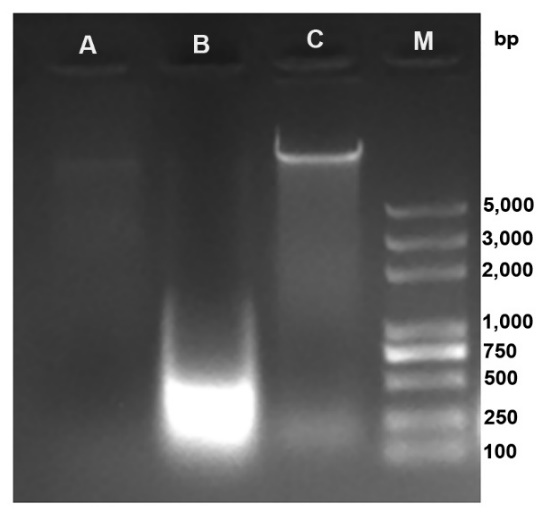
**

**Supplementary Figure 2.** Determination of nucleic acids in OMVs by agarose gel electrophoresis. Lane A, nucleic acids of OMVs of non-irradiated PAO1; lane B, nucleic acids of IOMVs of irradiated PAO1; lane C, genomic DNA of PAO1; lane M, DNA marker.


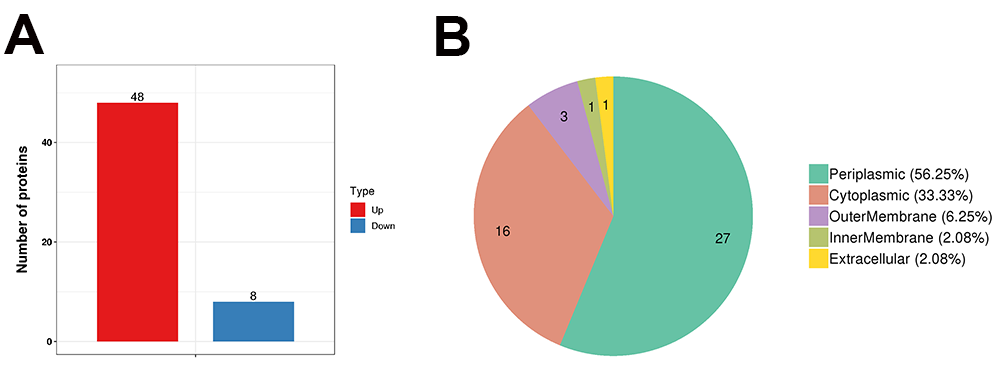


**Supplementary Figure 3.** (A) Histogram of the number distribution of the identified differentially expressed proteins. When p-value < 0.05, the change threshold of differential expression＞2 was significantly high abundance, and＜1/2 was significantly low abundance. (B) The distribution of the significantly high abundance proteins predicted according to subcellular localization.





**Supplementary Figure 4.** Analysis of the metabolic activity of PAO1 and Δ*recA* under X-ray irradiation using the Alamar Blue assay.

**References**

Hoang, T. T., Karkhoff-Schweizer, R. R., Kutchma, A. J., and Schweizer, H. P. (1998) A broad-host-range Flp-FRT recombination system for site-specific excision of chromosomally-located DNA sequences: application for isolation of unmarked *Pseudomonas aeruginosa* mutants. *Gene*. 212, 77-86. [doi: 10.1016/s0378-1119(98)00130-9](https://sci-hub.tw/10.1016/s0378-1119(98)00130-9)

Simon, R., Priefer, U., and Pühler, A. (1983) A Broad Host Range Mobilization System for In Vivo Genetic Engineering: Transposon Mutagenesis in Gram Negative Bacteria. *Bio/Technology*. 1, 784-791. [doi: 10.1038/nbt1183-784](https://sci-hub.im/10.1038/nbt1183-784)
